# Classification of processes involved in sharing individual participant data from clinical trials

**DOI:** 10.12688/f1000research.13789.2

**Published:** 2018-04-20

**Authors:** Christian Ohmann, Steve Canham, Rita Banzi, Wolfgang Kuchinke, Serena Battaglia

**Affiliations:** 1 European Clinical Research Infrastructure Network (ECRIN), Düsseldorf, 40477, Germany; 2 Canham Information Systems, Redhill, Surrey, RH1 6QH, UK; 3 Mario Negri Institute of Pharmacological Research, Milan, 20156, Italy; 4 Coordination Centre for Clinical Trials, Heinrich Heine University Dusseldorf, Dusseldorf, 40225, Germany; 5 European Clinical Research Infrastructure Network (ECRIN), Paris, 75013, France

**Keywords:** clinical trial, data sharing, individual participant data (IPD), process, business process model, generic framework

## Abstract

**Background:** In recent years, a cultural change in the handling of research data has resulted in the promotion of a culture of openness and an increased sharing of data. In the area of clinical trials, sharing of individual participant data involves a complex set of processes and the interaction of many actors and actions. Individual services and tools to support data sharing are becoming available, but what is missing is a detailed, structured and comprehensive list of processes and subprocesses involved and the tools and services needed.

**Methods**: Principles and recommendations from a published consensus document on data sharing were analysed in detail by a small expert group. Processes and subprocesses involved in data sharing were identified and linked to actors and possible supporting services and tools. Definitions adapted from the business process model and notation (BPMN) were applied in the analysis.

**Results:** A detailed and comprehensive tabulation of individual processes and subprocesses involved in data sharing, structured according to 9 main processes, is provided. Possible tools and services to support these processes are identified and grouped according to the major type of support.

**Conclusions:** The identification of the individual processes and subprocesses and supporting tools and services, is a first step towards development of a generic framework or architecture for the sharing of data from clinical trials. Such a framework is needed to provide an overview of how the various actors, research processes and services could interact to form a sustainable system for data sharing.

## Abbreviations

AAI, Authentication and Authorisation Infrastructure; API, Application Programming Interface; ATT, The Open Science and Research Initiative; BPMN, Business Process Model and Notation; BRIDG, Biomedical Research Integrated Domain Group; CDISC CDASH, Clinical Data Interchange Standards Consortium - Clinical Data Acquisition Standards Harmonization; CDISC ODM, Clinical Data Interchange Standards Consortium - Operational Data Model CDISC SDM, Clinical Data Interchange Standards Consortium - Study Design Model; COMET, Core Outcome Measures in Effectiveness Trials; CORBEL, Coordinated Research Infrastructures Building Enduring Life-science Services; CRUK, Cancer Research UK; DOI, Digital Object Identifier; ECRIN, European Clinical Research Infrastructure Network; EMA, European Medicines Agency; ICMJE, International Committee of Medical Journal Editors; ID, Identity; IPD, Individual Participant Data; IT, Information Technology; MRC, Medical Research Council (UK); PCROM, Primary Care Research Object Model; QA, Quality Assurance; UK, United Kingdom; UKCRC, UK Clinical Research Consortium; US, United States; WHO, World Health Organization

## Introduction

In recent years, many scientific organisations, funders and initiatives have expressed their commitment to more open scientific research. This cultural shift has been extended to also include clinical research and clinical trials in particular. Today, the results of clinical trials are increasingly considered as a public good, and access to the individual participant data (IPD) generated by those trials is seen as part of a fundamental right to health data (see Research Councils UK principles on data policy). At the same time, any release of data must include mechanisms to maintain the privacy of the trial participants, and properly recognise the work of the researchers who initially generated the data.

To support the sharing of IPD in clinical trials, several organisations have developed generic principles, guidance and practical recommendations for implementation in recent years (e.g. the Institute of Medicine report in the US ^1^, the Nordic Trial Alliance Working Group on Transparency and Registration for the Nordic countries ^2^, the good practice principles for sharing IPD from publicly funded trials by MRC, UKCRC, CRUK and Wellcome, in the UK ^3, 4^, the guide to publishing and sharing sensitive data for Australia ^5^ and the recommendations of the International Committee of Medical Journal Editors (ICMJE, see ICMJE recommendations on clinical trials). Within the EU Horizon 2020 funded project CORBEL (Coordinated Research Infrastructures Building Enduring Life-science Services) and coordinated by the European Clinical Research Infrastructure Network (ECRIN), an interdisciplinary and international stakeholder taskforce reached a detailed consensus on principles and recommendations for data sharing of clinical trial data ^6^. That document was taken as the starting point for the current paper.

Data sharing of IPD from clinical trials can be complex and will often involve the interaction of many actors. At present only limited documentary support is available, (e.g. templates for data sharing plans, data transfer and data use agreements), and this is scattered and thus not always easy to find. In addition, although some IT-tools and services are available to give support for individual tasks in the process of data sharing (e.g. for de-identification service for datasets; see Electronic Health Information Laboratory page on de-identification software) or an ID-generation service for study objects), these are again difficult to discover and their quality is not easy to assess. Additional complexity stems from the very heterogeneous set of repositories that are available for storage of IPD (see Registry of Research Data Repositories). There are general scientific repositories, repositories dedicated specifically to clinical research, repositories specialising a specific disease area and institution-specific repositories. Thus, although fragments of infrastructure are available to support sharing of IPD from clinical trials, the various services and tools are scattered and a global vision of how all these components should interact and interoperate does not currently exist.

Fundamentally, what is still missing is a generic framework or architecture for data sharing that could be used for modelling, describing, and designing operations, data requirements, IT-systems and technological solutions (see Open Group TOGAF® framework). Such a framework would link structural concepts (e.g. actors) with behavioural concepts (e.g. processes supported by services) and give an overview of how these could interact to form a complete system for data sharing of IPD. As a first step in creating such a general framework, we set out to identify various processes and subprocesses that could be involved and then provide a listing and classification of the tools and services that could usefully support those processes. It was not intended at this stage, to provide tools themselves (e.g. guidelines, examples, templates, IT-systems). This work is seen, however, as a necessary preparatory step for identifying and/or generating tools in a later stage of the CORBEL project.

## Methods

In this study, a semi-formal collaborative small group decision-making approach was used to derive and then critique the list of processes and subprocesses involved in data sharing. The work is non-quantitative and we have therefore applied elements of the COREQ guidelines for qualitative research, as applicable in the following discussion of methods.

### Credentials and experience of authors

CO, RB, SC and SB were members of the core team that coordinated the H2020 CORBEL working task on sharing of individual participant data from clinical trials lead by ECRIN. The team coordinated a consensus exercise of the multi-stakeholder taskforce and drafted the final report on ”Sharing and reuse of individual participant data from clinical trials: principles and recommendations” ^6^. WK was one of the experts within the multi-stakeholder taskforce.

The authors have different background and expertise, but all have a longstanding practical experience with clinical trials. CO has a PhD in mathematics and was head of an academic clinical trial unit with a focus on biostatistics and IT-support of trials; SC has an MSc in information systems and he is an expert in data management and IT systems for clinical trials, RB is a clinical pharmacologist with an expertise in clinical trial and evidence synthesis methodology; WK has a PhD in molecular genetics with education in clinical pharmacology and he is an expert of information science; SB has a PhD in biological sciences and is the project manager responsible for the CORBEL project for ECRIN.

Using a multi-stakeholder group of 40+ international experts, and a formal consensus building process, an overarching framework for IPD sharing and reuse was developed in the CORBEL project. That process was co-ordinated by and involved the extensive participation of the core team. The document produced covers all stages of the data sharing life cycle and is highly structured, with 7 main topics, 10 principles assigned to these topics and 50 specific recommendations, making the analysis of the processes and subprocesses involved in IPD sharing relatively straightforward. This process analysis can be considered a first step in translating these CORBEL’s principles and recommendations into actionable strategies, leading to implementation guidelines and the supporting services required for successful data sharing projects.

### Rationale for data collection

Other work on the sharing of IPD from clinical trials has usually been embedded in a geographical/national context (eg, US, Nordic countries, UK), or centred on a specific stakeholder group (eg. Pharma) or focused on a specific subset of clinical trial data (e.g. published data). Due to the heterogeneity of the different documents, it was decided not to attempt a systematic review. Instead these and other documents were taken into consideration in the initial CORBEL consensus exercise and, as a consequence, in the final report ^6^. Within this report we provided up to date, precise, broadly based and workable recommendations supporting data sharing from clinical trials. The report was generic though focus of the report was on non-commercial trials, a European origin and the perspective of the researcher. The CORBEL report provided the basis for this study ^6^.

### Limitations of the initial CORBEL consensus exercise

A limitation of the initial study was that the consensus building exercise was largely based on experience and opinions, and members of the task force may not have been fully representative of the research community. The other major issue is that the recommendations need to be implemented and tested in practice, and their feasibility and usability explored.

### Methodological approach

The basic concepts and definitions were adapted from the business process model and notation (BPMN) and applied to our analysis. Recommendations and principles from the data sharing consensus document were analysed in detail and individual processes and subprocesses identified and linked to actors and possible services and tools by a small group of experts (CO, SC, RB, WK, SB). The decision-making process was based on a facilitator (CO) providing initial and updated versions of the document and iterative rounds of written feedback from the team members. The process was continued until final agreement was achieved. The process took place between October 2017 and January 2018, four different versions were provided and approved in sequential order (24 November 2017, 7 and 11 December 2017, 15 January 2018). Due to the good relationship between the team members and long-term involvement in common projects, a comprehensive and detailed point of reference, the consensus document, and clear objectives with milestones and time lines, agreement could be achieved by the team without applying a normative model of decision-making. The protocol for the qualitative analysis of the processes was not registered.

***Definitions*.** The following definitions were adapted from the business process model and notation (BPMN) and applied to our analysis (see Object Management Group page, 7):

***Process:***                  A sequence or flow of activities in an organization with the objective of carrying out work (see Object Management Group page).

***Subprocess:***              A process that is included within another process (see Object Management Group page)

***Actor:***                  Some person or organization taking part in day-to-day business activity (see Object Management Group page)

***Service:***                  A service is a functional business entity that fulfils a particular requirement (see Open Science and Research framework)

In this study, processes may relate to different organisations and business goals, e.g. the various activities of the data generators, data storage managers and secondary users all represent *different* business processes, operating at different times by different actors.

Actors are belonging to or have a relationship with the clinical trial arena. Actors include: investigators, trial unit heads, QA-staff, senior data management and IT-staff, trial unit operational managers, statisticians, sponsors, trial management team, specialist agencies, repository managers, analysis environment providers, secondary users of data, data use advisory panel, research infrastructures, journal publishers, patient representatives, and funders. Definitions of actors have been taken from the glossary in the consensus document ^6^ and some from the CDISC-glossary.

Services and tools may be relatively non-technical (e.g. providing information, example materials, template policies and procedures, assessment criteria, metadata, and infrastructure specifications) or technical, i.e. information technology based. For the most part, the IT required is seen as relatively well established (e.g. webpages, web-based information systems) and already available (though would normally need specific organisation and application). A few services and tools may require specialist software development (e.g. development of an analysis environment, developing systems to support metadata repositories).

For graphical illustration, the BPMN approach was used. In BPMN, a process is depicted as a graph of flow elements, which are a set of activities, events, gateways, and sequence flow that adhere to a finite set of execution semantics. The usual BMBP notation and symbols were taken (event, activity, gateway, connections, swim lane) (see Object Management Group page). In this publication, BPMN is used only to give a high-level overview of the relation between the main processes. We may use the same notation in the future to ‘drill down’ into individual processes to provide a more detailed graphical representation.

## Results

From the analysis of the consensus document 9 **groups of processes** involved in sharing of IPD were identified. These were concerned with:

1. Preparation for data sharing, in general (3)

2. Plan for data sharing, in the context of a specific trial (5)

3. Preparation of data for sharing, after data collected (3)

4. Transferring data objects to an external repository (2)

5. Repository data and access management (6)

6. Access to individual participant data and associated data objects (2)

7. Discovering the data objects available (5)

8. Publishing results of re-use (1)

9. Monitoring data sharing (2)

**Figure 1.**
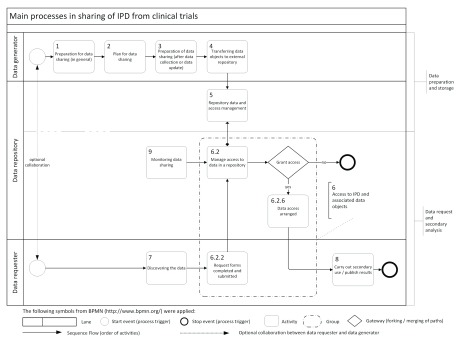
Overview on the main processes in sharing of IPD.

The numbers in brackets refer to the number of distinct processes identified within each group. Group 1 to 5 can be summarized under the heading “Data preparation and storage”, and 6–9 under the heading “Data request and secondary analysis”. The relationship between these major process groups is presented in Figure 1.

Almost all of the 29 processes were broken down further to 2 or 3 subprocesses, occasionally more and each subprocess was linked to the main actors involved and possible services and / or tools. As result a detailed and comprehensive list of the individual activity involved in data sharing is provided by Table 1.

**Table 1.** Listing of processes, actors and possible services/tools in sharing of IPD from clinical trials.

Process	Subprocess	Main Actors	Possible Services/Tools
1. Preparation for data sharing, *in general*			
1.1 Learn about individual participant data (IPD) and data object sharing ^1^.	1.1.1 Learn about policies, requirements, implications, options, resources, etc.	Investigators, trials unit ^2^ heads, operational managers, patient groups	Education service (web pages, videos, courses, texts etc.)
	1.1.2 Become aware of repositories available for data sharing, features, pros and cons, costs, etc.	Investigators, trials unit heads, operational managers	Web based information sources on repositories, published surveys, repository quality assessments
1.2 Develop local SOPs and related quality documents supporting aspects of IPD and data object sharing	1.2.1 Develop procedures governing data sharing planning and procedures within a trial.	Trials unit heads, QA staff, operational managers, senior data management and IT staff	Example SOPs and proformas
	1.2.2 Develop procedures and libraries to promote the use of data standards in database and metadata design.	Trials unit operational managers, statisticians, senior data management and IT staff	Links to standards and associated resources. Example local procedures Libraries of re-usable components
1.3 Clarify and integrate own institution’s requirements for data sharing	1.3.1 Clarify / agree with relevant university, hospital (etc.) any policies and requirements (e.g. use of local data repositories) concerning data sharing.	Trials unit heads, QA staff, operational managers, equivalent staff in parent organisation(s)	
	1.3.2 Integrate any institutional requirements into local SOPs and procedures.	Trials unit heads, QA staff, operational managers	
2. Plan for data sharing, *in the context of a specific trial*			
2.1 Decide the strategy for data sharing for this trial.	2.1.1 Explore options for data sharing (considering datasets, timeframe, likely de- identification required, planned journal (s), likely repository, costs, etc.)	Sponsors, with trial management team and network of investigators	Checklist of issues that need to be considered, with supporting material, option descriptions
	2.1.2 Check funder/sponsor requirements for data sharing	Trial management team, funder and / or sponsor staff	Classification of legal responsibilities of different parties involved
	2.1.3 Decide the strategy and specific actions required for data sharingin this trial	Sponsors, with trial management team	Checklist of issues that need to be considered, with supporting material, option descriptions
2.2 Document the strategy for data sharing for this trial in trial documents	2.2.1 Incorporate data sharing summary in section of the protocol	Trial management team	Example protocol sections
	2.2.2 Incorporate data sharing details within the data management plan	Trial management team, data management / sharing specialists	Example DMP sections with supporting material
	2.2.3 Incorporate data sharing summary within trial registration data	Trial management team	Example registry data sections
2.3 Incorporate information on data sharing plan into participant documents of clinical trials	2.3.1 Summarise and explain data sharing plan in patient information sheets.	Trial management team, patient groups and representatives	Guidance on legislation framework – demonstration material, templates, examples
	2.3.2 Include request for broad consent for data sharing in informed consent documents.	Trial management team, patient groups and representatives	Demonstration material, templates, examples
2.4 Check and align data sharing plans of collaborators who are also generating data.	2.4.1 Ensure any plans to publish collaborators‘ data (e.g. lab data) are compatible with plans for clinical IPD sharing	Trial management team	Examples of possible issues (e.g. with expectations of publishing lab data, increased re-identification risk)
	2.4.2 Ensure all collaborators have contributed to and have agreed to data sharing plans.	Trial management team	Examples of possible processes, policies, to agree and document data sharing across collaborators.
2.5 Ensure that data and metadata standards have been used as far as possible in database design		Trial management team	Links to standards and associated resources. Libraries of re-usable components
3. Preparation of data for sharing, after data collected			
3.1 Confirm strategy for data preparation for sharing, including timelines	3.1.1 Decide if (further) pseudonymisation or anonymisation required.	Trial data management and IT staff	Guidance on interpretation of legal requirements in different jurisdictions.
	3.1.2 Assess risk of re-identification with existing datasets. Confirm types and level of de- identification required.	Trial data management and IT staff, specialist de- identification agencies	De-identification/anonymisation service for datasets
3.2 Carry out strategy for data preparation	3.2.1 De-identify, and pseudo-anonymise or anonymise dataset for data sharing	Trial data management and IT staff, specialist de- identification agencies	De-identification/anonymisation service for datasets
	3.2.2 Check that analyses still function for de- identified data and document any discrepancies	Statisticians and data managers	
	3.2.3 Generate / transform, and check, descriptive metadata for the datasets prepared for sharing	Trial data management and IT staff	
	3.2.4 Select file formats for long term storage of data and metadata and transform if necessary	Trial data management and It staff	File formats recognized as standard
3.3 Document data preparation process	3.3.1 Assess and document risk of re- identification with revised datasets	Trial data management and IT staff, specialist de- identification agencies	De-identification/anonymisation service for datasets. template Data Protection Impact Assessments.
	3.3.2 Incorporate record of data preparation and risk assessments within metadata	Trial data management and IT staff	Metadata scheme for describing de- identification and data preparation processes
4. Transferring data objects to external repository			
4.1 Select repository (or confirm earlier repository selection)	4.1.1 Explore repository features, management, access options, costs, certification	Sponsors with trial management team	Data repository identification service including assessment against quality criteria, standards, certification process for repositories
4.2 Transfer the datasets under a formal data transfer agreement	4.2.1 Agree on access regime, data sharing decision processes, assignment of responsibilities including data controller role	Sponsors with trial management team	Checklists to support data transfer agreement
	4.2.2 Agree on responsibilities for generating discoverability and provenance metadata	Sponsors with trial management team	Checklists to support data transfer agreement
	4.2.3 Draw up and agree data transfer agreement, including provision if repository disappears	Sponsors with trial management team	Tools for generating data transfer agreement
	4.2.4 Apply discoverability and provenance metadata to datasets and transfer data	Trial data management and IT staff and/or repository staff	Metadata schemas for data object discoverability; tools for their application
5. Repository data and access management			
5.1 Maintain highly granular access control to IPD	5.1.1 Maintain access control that allows individual files to be designated as either a) publicly available, without user identification, to download or simply view. b) available only to *self-identified* named individuals, to download or simply view (may be managed on a group basis). c) available only to named individuals, *as identified by data controllers*, to download or simply view (may be managed on a group basis).	Repository managers	Authentication and authorisation tools, logging services
	5.1.2 Maintain 2-factor authentication, as required, with either (b) or (c) from 5.1.1.	Repository managers	Authentication and authorisation tools with 2 factor authentication (see ^4^ at the bottom of Table 1)
5.2 Maintain mechanisms to set up and apply authentication and authorization measures	5.2.1 Provide web based forms that allow users to provide details about themselves, with some degree of validation (e.g. email confirmation, cross reference to other AAI architectures).	Repository managers	Authentication and authorisation tools, validation mechanisms
	5.2.2 Provide appropriate log-in pages, with password management	Repository managers	Authentication and authorisation tools
5.3 Where there is a demand, provide a protected analysis environment	5.3.1 Allow datasets (including, possibly some imported from other repositories) to be identified and requested for a designated analysis environment	Repository managers, analysis environment providers	The analysis environment itself, including data import and logging tools
	5.3.2 Provide viewing, analysis and recording tools, while preventing download of the data.	Repository managers, analysis environment providers	Analysis tools and services, logging tools
	5.3.3 Provide workflow recording tools and documentation of the whole process, including the closure / teardown of the specific analysis environment.	Repository managers, analysis environment providers	Workflow recording tools, logging tools
5.4 Supply discovery data for IPD and data objects on a regular basis to metadata repositories	5.4.1 Liaise with metadata repositories to agree a metadata schema that conforms with, or can be mapped to, a general schema for discovery metadata	Repository managers	Schema for discovery metadata
	5.4.2 Allow regular (e.g. nightly) harvesting of metadata, through an API.	Repository managers	API for making metadata available from each repository
5.5 Facilitate data access according to prior Data Transfer Agreement	5.5.1 Establish a Data Access Committee, for data that requires this type of controlled access, which can process and filter requests, and recommend or take decision on data release.	Repository managers, Data Access Committee members	Guidelines for terms of reference / functioning of Data Access Committees; mechanisms for recording and publication of Access Committee decisions
	5.5.2 For data that requires them, create and post data request forms for users to complete.	Repository managers	Template and example data request forms
	5.5.3 For data that requires them, create templates that allow potential users to see the information they will need to provide, and the conditions to which they will need to conform.	Repository managers	Template and example data use agreements (may be starting points for negotiated, specific, agreements)
5.6 Provide usage and status reports to data depositors	5.6.1 Provide regular (e.g. quarterly) reports on access and / or requests made, by whom, actions taken and reasons given, back to the data generators and / or controllers	Repository managers	Report services maintained by repository managers
	5.6.2 Provide regular (e.g. annual) reports on management of data security and integrity, changes in infrastructure, funding and organisation, etc., to all users	Repository managers	Report services maintained by repository managers
6 Managing access to individual participant data and associated data objects			
6.1 Manage direct responses to the sponsors or coordinating investigators, in case no legal sponsor is available (data not yet in a repository)	6.1.1 Decide upon the possibility, in legal terms, of making the data available to others at all.	Sponsors and trial management team	Guidance on interpretation of legal requirements in different jurisdictions, for different levels of consent
	6.1.2 Assess the reasonableness of the request and the ability of the requesters to draw sensible conclusions	Sponsors and trial management team	
	6.1.3 Assess the costs of de-identifying the data, preparing metadata, etc.	Sponsors and trial management team	Data on costs in data preparation exercises
	6.1.4 Make a final decision as to whether to share the data with the requester.	Sponsors and trial management team	
	6.1.5 Draw up a data use agreement and transfer the data under its terms	Sponsors and trial management team	Example data use agreements
6.2 Manage access to data in a repository (if access requests individually reviewed)	6.2.1 Repository makes appropriate request forms available on-line	Repository managers	Available forms on line (see 5.5)
	6.2.2 Request forms completed and submitted (on or off-line)	Secondary users	
	6.2.3 (If stipulated in data transfer agreement) Request passed to advisory panel for assessment and recommendation, otherwise to data controllers	Sponsors or advisory panel	
	6.2.4 Decision to allow request made, by Data Access Committee if stipulated in data transfer agreement, otherwise by data controllers	Sponsors or Data Access Committee, or repository managers	Guidelines for terms of reference / functioning of Data Access Committees
	6.2.5 If positive decision, data use agreement drawn up and agreed	Sponsors or advisory panel, repository managers, secondary users	Example data use agreements
	6.2.6 Data access arranged after liaison with repository managers	Sponsors or advisory panel, repository managers	Pipeline for quick processing of access change requests
	6.2.7 Access request and decision documented	Sponsors or advisory panel, repository managers	Recording systems for request and decision
7. Discovering the data			
7.1 Agree a common discovery metadata standard		Repository managers, metadata repository managers	The metadata scheme itself
7.2 Agree and implement an ID generation scheme for data objects	7.2.1 Develop, and cost a mechanism for generating persistent IDs for data objects.	Repository managers, metadata repository managers	Existing ID supply mechanisms, especially DOIs
	7.2.2 Implement the process for generating persistent IDs (e.g. DOIs) for data objects.	Trial teams, repository and metadata repository managers	The ID generation mechanism itself
7.3 Agree and implement an ID generation scheme for clinical studies	7.3.1 Use existing (multiple) study identifiers as far as possible	Repository managers, metadata repository managers, (etc!)	Existing IDs, from registries, sponsors, funders etc.
	7.3.2 Attempt to develop an ID generation and / or assignment process for *all* clinical studies	Repository, metadata repository and registry managers, WHO, etc!	Existing ID supply mechanisms, especially registry IDs
7.4 Collect metadata together into a public metadata repository, under a single portal	7.4.1 Collect existing metadata samples and sources into a prototype metadata repository	Metadata repository managers	The metadata scheme from 7.1, the ID schemes from 7.2, 7.3;
	7.4.2 Maintain the metadata by arranging regular harvesting (e.g. nightly, using API and metadata scheme)	Metadata repository managers	The metadata scheme from 7.1, the ID schemes from 7.2, 7.3; Metadata harvesting tools
	7.4.3 Develop a single portal for searching through the metadata	Metadata repository managers	
	7.4.4 Federate additional metadata sources under the same portal	Metadata repository managers	
7.5 Search for the data objects concerned with a trial or clinical study		Secondary users	Search tools using study identifiers, name, and / or object identifiers. Receive data on location and access details.
8. Publishing results of re-use			
8.1 Carry out secondary use and publish results	8.1.1 Publish re-analysis, preferably open (e.g. peer reviewed journal)	Secondary users	
	8.1.2 If successful, ensure proper citation of data and credit to data generators.	Secondary users	Agreed schemes for citation and credit for data
	8.1.3 Whether or not published in a journal, publish summary results and relevant datasets – usually in source repository.	Secondary users, repository managers	
	8.1.4 Apply metadata to new data objects, ensure harvesting into metadata system.	Secondary users	Metadata scheme for discoverability
9. Monitoring data sharing			
9.1 Monitor data sharing activity	9.1.1 Gather and disseminate data on data requests (where explicit requests are required).	Repository managers	Web site on which to display collected data
	9.1.2 Gather and disseminate data on reasons for request refusal (where explicit requests are required).	Repository managers	Web site on which to display collected data
	9.1.3 Gather and disseminate data on data accesses, downloads etc.	Repository managers	Web site on which to display collected data
9.2 Monitor output and consequences of data sharing	9.2.1 Attempt to monitor output of data sharing activity and associated research (papers, datasets etc.).	Publishers, funders	Web site on which to display collected data
	9.2.2 Attempt to monitor level and outcome of disputes that may occur after data sharing and re-analysis	Publishers, funders, individual researchers	Published papers
	9.2.3 Attempt to monitor changing attitudes towards data sharing in clinical research	Individual researchers	Published papers

^1^ **Data objects**: any discrete packages of data in an electronic form – whether that data is textual, numerical, a structured dataset, an image, film clip, (etc.) in form. They are each a file, as that term is used within computer systems, and are named, at least within their source file system. In the context of clinical research and data sharing, data objects can include electronic forms of protocols, journal papers, patient consent forms, analysis plans, and any other documents associated with the study, as well as datasets representing different portions and types of the data generated, and the metadata describing that data. ^2^ **SOP**: Standard Operating Procedure – A controlled document, explicitly versioned, reviewed and approved, that outlines the roles and responsibilities involved in a particular task and / or workflow, and the subtasks, deliverables and associated documentation required. SOPs may be supplemented by more detailed ‘work instructions’, that may relate to using one or more specific systems. ^3^ **Authentication**: The process of ensuring that a person or system that is trying to access a system is who they say (it says) they are. With a person, authentication is by provision of one or more of something only they should know (e.g. a password), or should have (e.g. a card or fob), or can show (e.g. fingerprint, iris pattern). With a system it is more often by provision of a secret token (in effect a machine password), often derived from public key cryptography. ^4^ **Two factor authentication**: The simultaneous use, by a person, of two of the three authentication methods described above. ^5^ **Authorisation**: The process of giving an authenticated entity the rights to access particular subsets of data and/or to carry out particular functions within a system. It is usually carried out by assigning user entities to roles and to groups that together define the access allowed.

In Table 2, the possible services and / or tools associated with this activity are grouped according to major types of support, with a reference to the subprocesses where they may provide support. As the table illustrates, these tools and services fall into 6 (overlapping) categories:

1. Providing general background material

2. Locator services (for resources for data sharing, and / or to support data standards)

3. Example documents and templates

4. Services (e.g. to de-identify data, assign IDs, provide metadata, evaluate repositories)

5. Frameworks and guidance (e.g. metadata schemas, citation systems, checklists)

6. Tools (IT based, e.g. APIs to harvest repository contents, tools to assign metadata)

**Table 2.** Classification and description of possible tools/services to support processes in sharing IPD from clinical trials.

Type of service/tool	Description/comments	Reference to process ( Table 1)
**1. Providing general background material**		
Providing general background material	Collection of relevant resources about data sharing *in general* – e.g. • Links to papers and relevant policy documents from an annotated bibliography, • Summary documents (e.g. built around recent consensus paper) and web pages • Glossary of terms • Links to general educational and training resources provided elsewhere • Courses, webinars, books using materials above • Meetings, conference sessions looking at aspects of data sharing and related topics • Advice to citizens, ethics committees	1.1.1
**2. Locator services**		
List of general resources to support data sharing	Annotated links to web sites that provide (for example) … • Data on repositories for storage of datasets and other data objects (see ^1^ at the bottom of Table 1), and their facilities, terms of service etc. • Data on services to aid in de-identification • Information on the applicable legal framework(s) • Links to model agreements templates that can be adapted to meet the particular circumstances of data sharing projects.	1.1.2
List of resources to support greater use of data standards	Annotated links to • Repositories of standard data items, e.g. within CDISC’s CDASH, CFAST. • Repositories of standard data instruments, e.g. CDISC QRS (questionnaires, ratings and scales) • Metadata schemes • Core outcome sets (e.g. COMET)	1.2.2, 2.5
**3. Example documents**		
Example documents supporting data sharing processes	• Example SOPs, (see ^2^ at the bottom of Table 1), • Supporting relevant checklists, forms Covering all aspects of data sharing, e.g. • During study preparation, or as part of long term data management, in the context of pre-defined collaborations, or when handling requests for access. • Use of data standards in study design • Use of metadata for data description, data object discovery • Examples of data sharing policies (universities, research institute) • Examples of data sharing requests from funders or journals	1.2.1, 2.1, 2.4.1, 2.4.2
Example data sharing documents (for trial set up)	Examples of possible • Sections of a protocol • Sections of a Data Management Plan • Trial Registry sections • Participant information sheets • Consent forms • Proformas, for agreements with collaborators • Proformas, for using lab and genetic data All dealing with aspects of *planning* for data sharing and publication plans, available as a central resource. These could then be used / adapted in the context of individual trials.	2.2.1, 2.2.2, 2.2.3, 2.3.1, 2.3.2, 2.4.1
Example data sharing documents (for data transfer)	Examples / templates of possible • Data transfer agreements • Relevant sections of a Data Management Plan • Checklists for the data transfer process	4.2.1, 4.2.3
Example data sharing documents (for data re-use)	Examples of • Data request forms • Data use agreements • Checklists to support the development of a data use agreement	5.5.3, 6.1.5
**4. Services**		
De-identification / anonymisation service for datasets	There are four possible services here – • Resources that allow trials units to develop their own de-identification/anonymisation processes (if compliant with legal considerations). • Consultancy input to advise on de-identification in the context of a particular trial • Services that carry out and document a de-identification process on behalf of the sponsor / trials unit • Service for assessment of risk of re-identification	3.1.2, 3.2.1, 3.3.1, 3.3.2
Descriptive metadata services for datasets	To be useful (easily searchable, comparable etc.) the descriptive metadata of the data needs to be in a standard format, or one of a few recognised standard formats (e.g. CDISC ODM). Mechanisms and / or services to convert proprietary metadata descriptions into such a format could therefore be useful when required.	3.2.3, 3.3.2
Assessment / certification service for data repositories	Provision of a set of standards, that can be used to assess the suitability of any repository as a location for data object storage, would act as a useful guide to the potential users of those repositories. The further application of such standards within a certification scheme	4.1.1, 4.2.1
An ID assignment mechanism for data objects	An ID (e.g. doi) generation service is required for all stored data objects.	7.2.1, 7.2.2
A common pipeline for processing access requests	With the possibility of many different data repositories emerging storing clinical datasets, there is potential advantage from making the application, review, decision making process for each very similar (e.g. using common application proformas) or even managing those processes together, e.g. with a common expert advisory board. This could ultimately create a common ‘request pipeline’.	6.2.6
Recording and reporting systems for data access requests and episodes	Reports that could be provided by repositories include • Level and type of data object deposition • The types of data access arrangements in place • Numbers and types of access requests • The decisions reached and reasons for rejections Data objects generated as a result of data re-use.	5.6.1, 6.2.7, 9.1.1, 9.1.2, 9.1.3
Provision of a prototype metadata repository	A metadata repository, (or a portal linked to multiple such repositories) with discovery metadata for clinical trial data objects, is seen as a fundamental requirement if data sharing is going to work efficiently.	7.4.1, 7.4.2, 7.4.3, 7.4.4, 7.5
Service for provision of a secure analysis environment	Based on tools to provide an analysis environment for in-situ work (see below).	5.3.1, 5.3.2, 5.3.3
**5. Frameworks and guidance**		
The development of a discovery metadata schema	Agreement is needed on a common discovery metadata standard that can link data objects to studies and that can describe the access mechanisms associated with each. Proposals have been made, based on an existing scheme (DataCite) but need further development.	4.2.4, 5.4.1, 5.4.2, 7.1
The development of an agreed scheme for citation of re-use	There needs to be a universally recognised scheme that will allow fair credit for the re-use of data, in terms of academic citation and recognition.	8.1.2
Legal and regulatory framework	As the legal and regulatory environment continues to evolve, there will be an ongoing need to clarify the legal responsibilities of the major parties involved in data sharing by update relevant resources (e.g. templates, legal issues database, procedures). and keep researchers and data managers informed of any relevant changes in laws, policies, and regulations Such a service could usefully be a central resource. It could not be a legal service as such (i.e. answering specific questions) but it could provide a general framework for guidance.	2.1.2, 2.3.1, 3.1.1, 6.1.1
Checklist to decide the strategy for data sharing	Checklist of issues to be considered of data sharing, with supporting material, option descriptions	2.1.1, 2.1.2, 2.1.3
Checklist to support specification of agreements	Checklist to support development of data transfer agreement/data use agreement	4.2.1, 4.2.2
Manual to establish boards overseeing data sharing	Manual for advisory panel/board	5.5.1, 6.2.4
**6. Tools**		
Tools to support the application of discovery metadata scheme	A tool is required to allow the easy application of the metadata schema used to characterize data objects, ideally by the object generators and if not by repository managers. This would likely take the shape of a set of web based forms, linked to a central repository.	4.2.4, 8.1.4
Tools for de-identification / anonymisation service for datasets	See de-identification / anonymisation service for datasets above,	3.1.2, 3.2.1, 3.3.1, 3.3.2
Authentication and authorisation systems for repository access (see ^3^ and ^5^ at the bottom of Table 1)	Highly granular access is needed (at the level of individual users / individual data objects) to support the variety of controlled access mechanisms likely to be required in repositories	5.1.1, 5.1.2, 5.2.1, 5.2.2
Provide an analysis environment for *in-situ* work	Interest has been expressed in a mechanism that allows data to be examined, re-analysed, aggregated etc. without being downloaded first, but instead kept within a secured, tailored, ‘analysis environment’, which also contains the analysis tools required. In fact several different types of tools would be required, for: • Environment creation (e.g. as a container) • Data import and logging • Authentication and authorisation • Analysis • Workflow recording • Environment destruction	5.3.1, 5.3.2, 5.3.3
APIs to access repository catalogue data (for metadata aggregation)	When discovery data is not (or has not been) directly transferred to a central repository using the tools described above, it will be necessary to try and ‘harvest’ metadata from data repositories on a regular basis. Using APIs that give access to the repository catalogues is a key part of that, and is much cheaper than trying to use ‘data mining’ techniques, e.g. natural language parsing on data object titles, to link data objects to studies.	5.4.1, 5.4.2
Tools for generation of data transfer agreements/data use agreements	Software tools supporting the development of data transfer/data use agreements.	4.2.3, 6.1.5, 6.2.5

## Discussion

Within the framework of the EU H2020 funded project CORBEL major issues associated with sharing of IPD were investigated and a consensus document on providing access to IPD from clinical trials was developed, using a broad interdisciplinary approach ^6^. The taskforce reached consensus on 10 principles and 50 recommendations, representing the fundamental requirements of any framework used for the sharing of clinical trials data. To support the adoption of the recommendations, adequate tools and services are needed to promote and support data sharing and re-use amongst researchers, adequately inform trial participants and protect their rights, and provide effective and efficient systems for preparing, storing, and accessing data. As a first step on the way to inventory existing tools/services, their quality and applicability for data sharing, a systematic analysis of processes and actors involved in data sharing was performed. The work done resulted in a systematic, structured and comprehensive list of processes and subprocesses that need to be supported to make data sharing a reality in the future. It is basic work against which existing tools and and services can be mapped, and allowing gaps in service provision to be identified. It is outside the scope of this paper to address issues about data sharing (e.g. recognition of the effort of the original researcher, self-identification of patients). This has been addressed in the CORBEL consensus exercise ^6^.

In the context of this work, we explored the possibility of generating a generic frameork for the sharing of IPD from clinical trials. As an example we considered the Framework for Open Science and Research by ATT (see Open Science and Research framework). This framework provides a general description of a desired architecture in a domain of open science, defining the key structural elements of the overall solution and describing their interactions, using an Enterprise Architecture (EA) approach. It can thereby give an overview of how various research processes, actors and services – including data, data structures, and IT-systems – could form an interoperable system in the ‘target’ open state. The work done in developing a framework for open science and research could be of major relevance for a similar model in the area of participant data sharing. At this stage, however, of identifying the processes and subprocesses involved, it was felt to be too early to develop a generic framework. It may be that this approach will be taken up again once there is confidence that the components for such a framework have been identified.

Nevertheless, we thought it useful to use a standardised terminology and notation for describing basic processes in data sharing. This will simplify the extension to a more generic and comprehensive framework at a later stage. As one approach, business modelling has been applied successfully in the health and health research area. It has been used, for example, to perform a requirements analysis of the barriers to conducting research linking of primary care, genetic and cancer data ^7^, to model the complexity of health and associated data flow in asthma ^8^ and to provide a generic architecture for a type 2 diabetes mellitus care system ^9^. We decided not to apply the full spectrum of business process modelling (BPMN), but to use only basic elements to give a notational and terminological basis for further work. More work is needed to explore the suitability and benefit of BPMN for a generic framework for data sharing.

Different models for clinical trials and clinical trials workflow already exist, such as the domain analysis model BRIDG ^10^, the study design model CDISC SDM ^11^ and the primary care information model PCROM ^12^. Any framework or model for data sharing needs to map or reference these clinical trial models, though none currently include the secondary use of data after the trial has completed. Although clinical trial processes and data sharing processes are distinct, they are clearly linked, and any comprehensive model needs to incorporate those linkages.

Many of the services and tools identified in this paper are non-technical but nevertheless may be of major importance, especially for data generators and data requestors. This includes templates and examples, checklists and guidance. For some of the processes specified in this paper IT-tools and services already exist and can be applied (e.g. de-identification tools and services, see Electronic Health Information Laboratory page on de-identification software), others are under development but need further work or an extension in scale (e.g. metadata repository for identifying clinical trial objects, ^13^). This work could also be used as input to an update of the EMA data sharing policy, currently discussing the possibility of sharing individual participant data (IPD) from clinical trials (see EMA page of clinical data publication policy). The next step is to perform a scan on the availability and suitability of services and tools for data sharing based on this work, with the involvement of stakeholders. We will summarize this information in a separate report.

## Data availability

The data referenced by this article are under copyright with the following copyright statement: Copyright: © 2018 Ohmann C et al.

Data associated with the article are available under the terms of the Creative Commons Zero "No rights reserved" data waiver (CC0 1.0 Public domain dedication). 

All data underlying the results are available as part of the article and no additional source data are required.
