# Supplementary material for: Classification of processes involved in sharing individual participant data from clinical trials
Source: F1000Res. 2018 Apr 20;7:138. Originally published 2018 Feb 1. [Version 2] doi: 10.12688/f1000research.13789.2 (PMC5861517; doi:10.12688/f1000research.13789.2)
Supplement: Peer review history

# Peer review: Referee response for version 2

**DOI:** 10.5256/f1000research.15913.r33332

**Published:** 2018-05-21

**Authors:** Matthew R. Sydes

**Affiliations:** 1 MRC Clinical Trials Unit at UCL, Institute of Clinical Trials and Methodology, University College London, London, UK

I appreciate the constructive responses from the authors and their revisionary steps. I accept that some key points are out of scope for this manuscript and I'd be happy to join discussions to consider them further.

 I have no further points to add at this stage.

I have read this submission. I believe that I have an appropriate level of expertise to confirm that it is of an acceptable scientific standard.

# Peer review: Referee response for version 2

**DOI:** 10.5256/f1000research.15913.r33331

**Published:** 2018-05-08

**Authors:** Matthias Löbe

**Affiliations:** 1 Institute for Medical Informatics, Statistics and Epidemiology (IMISE), Leipzig University, Leipzig, Germany

Thank you for the changes and explanations. Our comments have been fully taken into account.

I have read this submission. I believe that I have an appropriate level of expertise to confirm that it is of an acceptable scientific standard.

# Peer review: Referee response for version 2

**DOI:** 10.5256/f1000research.15913.r33333

**Published:** 2018-04-20

**Authors:** Florian Naudet

**Affiliations:** 1 CHU Rennes, Inserm, CIC 1414 (Centre d'Investigation Clinique de Rennes), University of Rennes 1, Rennes, France

No additional comment.

I have read this submission. I believe that I have an appropriate level of expertise to confirm that it is of an acceptable scientific standard.

# Peer review: Referee response for version 1

**DOI:** 10.5256/f1000research.14988.r31018

**Published:** 2018-03-20

**Authors:** Matthias Löbe

**Affiliations:** 1 Institute for Medical Informatics, Statistics and Epidemiology (IMISE), Leipzig University, Leipzig, Germany

In this paper, Ohmann et. al. perform a detailed analysis of steps required to share patient microdata from clinical trials with the research community. They provide a process diagram describing the workflow of preparing, transferring and maintaining the data and metadata to an external repository. The main part of the work consists of a comprehensive list of all sub-processes, the involved actors and services or tools. They also elaborate on scope and depth of the services or tools and give examples.

 The valuable contribution of this work lies in the sequential structuring of data sharing tasks. Especially study groups who want (or have to) actively provide data have a checklist at hand, which gives them the opportunity to assess each sub-task in its complexity and to put together suitable persons or teams for implementation. This prevents important stakeholders from being overlooked or partial steps from being insufficiently taken into account, particularly with regard to regulatory issues.

 The article focuses on aspects of data sharing in clinical trials, addressing a relevant problem of academic research, namely the long-term availability of research results in an environment that has only a limited lifespan due to project funding. It shows the complexity of the topic and every research group should already think about it during the project planning phase. Additionally, it is also relevant for other types of research projects, such as clinical registries, epidemiological cohorts or studies in health care research, with minor modifications.

 I particularly liked the fact that aspects of providing analysis environments were also addressed, e.g. with special Docker containers that bring the evaluation algorithms to the data instead of releasing data.

 The weak part of the paper is that even with a detailed listing of the sub-processes and the relevant tools, most researchers will find it difficult to design a concrete implementation strategy or to check whether the implementation meets the state of the art. Notes such as "Provide sample documents", "Assess risk of re-identification" or "Select suitable metadata schemas for object discovery" are simply too vague to be a real help. At this point, a knowledge base must be built up that provides researchers with concrete guidelines, implementation guidelines and example scenarios for successful projects.

 Points to address:

- The workflow in Figure 1 assumes that the data set is only imported once into an external repository. However, there are many scenarios in which data sets will have to be updated or extended, e.g. in long-running investigations where interim evaluations are already being carried out. Snapshots of shared data must be saved for verification purposes.
- Some years ago, there has been an EMA draft policy on publication and access to clinical-trial data [1]. I’m not sure about the current status but it would be interesting to include the effort in this paper.
- Page 6, section 2.3.2 “Include request for broad consent for data sharing in informed consent documents.” The term broad consent might require a more detailed definition, because in Germany consent is always contextual and without specific and the ethics committees are looking into this.
- Metadata (sections 2.5, 5.4, 7.1) should not be limited to semantics and discovery. Another important topic for metadata is provenance metadata (measurement conditions, data quality, algorithms for calculated data)

I have read this submission. I believe that I have an appropriate level of expertise to confirm that it is of an acceptable scientific standard, however I have significant reservations, as outlined above.

# Response

**Published:** 2018-04-16

**Authors:** Christian Ohmann

**Affiliations:** ECRIN, Germany

***Our answer in bold and italics***. 

In this paper, Ohmann et. al. perform a detailed analysis of steps required to share patient microdata from clinical trials with the research community. They provide a process diagram describing the workflow of preparing, transferring and maintaining the data and metadata to an external repository. The main part of the work consists of a comprehensive list of all sub-processes, the involved actors and services or tools. They also elaborate on scope and depth of the services or tools and give examples.

The valuable contribution of this work lies in the sequential structuring of data sharing tasks. Especially study groups who want (or have to) actively provide data have a checklist at hand, which gives them the opportunity to assess each sub-task in its complexity and to put together suitable persons or teams for implementation. This prevents important stakeholders from being overlooked or partial steps from being insufficiently taken into account, particularly with regard to regulatory issues.

The article focuses on aspects of data sharing in clinical trials, addressing a relevant problem of academic research, namely the long-term availability of research results in an environment that has only a limited lifespan due to project funding. It shows the complexity of the topic and every research group should already think about it during the project planning phase. Additionally, it is also relevant for other types of research projects, such as clinical registries, epidemiological cohorts or studies in health care research, with minor modifications.

I particularly liked the fact that aspects of providing analysis environments were also addressed, e.g. with special Docker containers that bring the evaluation algorithms to the data instead of releasing data.

The weak part of the paper is that even with a detailed listing of the sub-processes and the relevant tools, most researchers will find it difficult to design a concrete implementation strategy or to check whether the implementation meets the state of the art. Notes such as "Provide sample documents", "Assess risk of re-identification" or "Select suitable metadata schemas for object discovery" are simply too vague to be a real help. At this point, a knowledge base must be built up that provides researchers with concrete guidelines, implementation guidelines and example scenarios for successful projects.

***The purpose of the study was better explained at the end of the introduction. It was the objective to identify all processes/sub-processes involved in data sharing and to provide a classification of tools/services needed to support the processes. It is ground structuring work and it was not intended to provide specific help for data sharing (e.g. guidelines, examples).*** ***In a later stage of the CORBEL project concrete and speciifc tools/services to support data sharing will be made availalbe.***

Points to address:

- The workflow in Figure 1 assumes that the data set is only imported once into an external repository. However, there are many scenarios in which data sets will have to be updated or extended, e.g. in long-running investigations where interim evaluations are already being carried out. Snapshots of shared data must be saved for verification purposes.
***This is a relevant point and was included in the figure under 3) : Preparation of data sharing (after data collected or data update. ***
- Some years ago, there has been an EMA draft policy on publication and access to clinical-trial data [1]. I’m not sure about the current status but it would be interesting to include the effort in this paper.
***The EMA policy 70 is effective since January 2015 and applies to new drugs approved by the EMA after that date, thus only on a subset of trials testing pharmacological interventions.*** ***Moreover, the policy is only dealing with clinical study reports, i.e. aggregate data.  Currently, the EMA is discussing the possibility of sharing individual participant data (IPD) from clinical trials. One EMA expert was included in our consensus exercise and one author of the current paper (CO) was invited to attend an EMA-workshop on anonymisation, 30.11.-1.12.2017). This publication could be used as input to an update of the EMA data sharing policy. This comment is added to the discussion. ***
- Page 6, section 2.3.2 “Include request for broad consent for data sharing in informed consent documents.” The term broad consent might require a more detailed definition, because in Germany consent is always contextual and without specific and the ethics committees are looking into this.
***The concept of broad consent has been discussed in detail in the BMJ Open paper published by the group in 2017.and was not tackled in this manuscript. ***
- Metadata (sections 2.5, 5.4, 7.1) should not be limited to semantics and discovery. Another important topic for metadata is provenance metadata (measurement conditions, data quality, algorithms for calculated data)
***Yes, provenance data are very important and an essential part of the metadata.  We have added provenance metadata in 4.2.2 and 4.2.4. ***

# Peer review: Referee response for version 1

**DOI:** 10.5256/f1000research.14988.r31482

**Published:** 2018-03-19

**Authors:** Matthew R. Sydes

**Affiliations:** 1 MRC Clinical Trials Unit at UCL, Institute of Clinical Trials and Methodology, University College London, London, UK

This process-orientated manuscript covers a lot of ground in some detail. I have some specific comments:

**Major** 

1. Section: General
  Comment: The process of reaching these recommendations is unclear to me. Perhaps these are opinions? I don’t think there is primary evidence to underpin them. Should there be?
2. Section: General
  Comment: This is comprehensive, but also sets out a substantial burden on organisations. I wonder for what proportion of trials this work is proportionate effort.
3. Section: General
  Comment: This does not address my previous concerns about recognition of effort of the original researchers or issues about self-identification by patients, but perhaps that is outside of the scope of the paper. It would helpful to remind the reader that these are key, unresolved issues and point to places where they might be considered further.

** **


**Moderate **

1. Section: Table 1
  Text ref: "1.2 Clarify own institution’s requirements for data sharing"
  Comment: This is pretty vague. I don’t know how to use this row.
2. Section: Table 1
  Text ref: "2.1.2 Check funder requirements for data sharing"
  Comment: Which takes priority and when? 2.1.2 vs 1.2.
3. Section: Table 1
  Text ref: "2. Plan for data sharing, in the context of a specific trial"
  Comment: When should this be developed? 2.2.2 suggests before the protocol is finalised; but I suspect 2.2.2 would generally be done before 2.2.1. What is the ordering of the rows?
4. Section: Table 1
  Text ref: "3.1 Decide upon strategy for data preparation for sharing"
  Comment: 3.1.1 and 3.1.2 seem to be in the wrong order.
5. Section: Table 1
  Text ref: Section 3 or 4
  Comment: Somewhere, perhaps, one should advertise the timelines for making data available. It’s unlikely to be during the trial; how long after primary analyses? Useful to manage expectations?
6. Section: Table 1
  Text ref: "5.1 Maintain highly granular access control to IPD, that can be changed rapidly"
  Comment: Changed on what basis?
7. Section: Table 1
  Text ref: "5.5 Provide an expert advisory panel"
  Comment: Is this a Data Access Committee or something different? Is there independent membership?
8. Section: Table 1
  Text ref: "5.7 Provide data use agreement templates"
  Comment: Possibly wishful thinking. Agreements are never as straightforward as one might hope. Is this a suggestion for global templates, institution templates or trial templates?
9. Section: Table 1
  Text ref: "6.1.2 Assess the reasonableness of the request and the ability of the requesters to draw sensible conclusions"
  Comment: Where is the independence in this process? Is there a duty from the sponsor and TMG to work fairly? Who judges what is reasonable?
10. Section: Table 1
  Text ref :: "6.2.1 Repository makes appropriate request forms available on-line"
  Comment: Why? This will just encourage false positive submissions. Better for applicants to talk to the trial team before getting a form, so the applicant really understands whether the data set is suitable and timely. (Very often, it really won’t be.)
11. Section Table 1
  Text refL "7.2 Agree an ID generation scheme for data objects"
  Comment: Also, what if the same dataset is given to two separate people: does this get the same ID?
12. Section: Table 1
  Text ref: "8. Publishing results of re-use"
  Comment: Who checks that the secondary use of the data is done well?
13. Section: Table 1
  Text ref: "8. Publishing results of re-use"
  Comment: What to do if there is discrepancy in findings between original and subsequent findings? Could undermine trust. Probably needs rows about “dispute” resolution.
14. Section: Table 2
  Text ref: "2. Locator services. Locator service for data sharing resources"
  Comment: Will this be a familiar term to readers? I’m not sure what it means.

 

**Trivial/Minor **

1. Section: Table 1
  Comment: Would be quickly for each actor to find the role if this column was broken into separate columns, one per actor type, with the ticks for whether it is relevant.
2. Section: Table 1
  Text ref: "7.2 Agree an ID generation scheme for data objects"
  Comment: “Data objects” needs a clear definition before the table. Perhaps a Glossary with the Abbreviations?

I have read this submission. I believe that I have an appropriate level of expertise to confirm that it is of an acceptable scientific standard, however I have significant reservations, as outlined above.

# Response

**Published:** 2018-04-16

**Authors:** Christian Ohmann

**Affiliations:** ECRIN, Germany

***Response to  reviewer in bold and italics***

This process-orientated manuscript covers a lot of ground in some detail. I have some specific comments:

  

**Major** 

1. Section: GeneralComment: The process of reaching these recommendations is unclear to me. Perhaps these are opinions? I don’t think there is primary evidence to underpin them. Should there be?
***Principles and recommendations on data sharing were developed in the BMJ Open paper. In this study a framework based upon these principles and recommendations was proposed, characterising processes/suprocesses as well as tools/services needed for data sharing.  T*** ***he following methodological approach was followed. The basic concepts and definitions were adapted from the business process model and notation (BPMN) and applied to our analysis.*** ***Recommendations and principles from the data sharing consensus document were analysed in detail and individual processes/subprocesses identified and linked to actors and possible services/tools by a small group of experts (CO, SC, RB, WK, SB).  The decision-making process was based on a facilitator (CO) providing initial and updated versions of the document and iterative rounds of written feedback from the team members.  The process was continued until final agreement was achieved. The process took place between October 2017 and January 2018, four different versions were provided and approved in sequential order (24 November 2017, 7 and 11 December 2017, 15 January 2018).  Due to the good relationship between the team members and long-term involvement in common projects, a comprehensive and detailed point of reference, the consensus document, and clear objectives with milestones and time lines, agreement could be achieved by the team without applying a normative model of decision-making.  As suggested by another reviewer, this paper can be classified as qualitative research, although we applied a semi-formal collaborative small group decision-making approach and not formal methodology such as interviews or focus groups.*** ***We revised the methodological section of the manuscript and adapted it as much as possible to the COREQ guidelines for qualitative research.***
2. Section: GeneralComment: This is comprehensive, but also sets out a substantial burden on organisations. I wonder for what proportion of trials this work is proportionate effort.
***This is difficult to estimate. The empirical assessment of the benefit of data sharing in comparison to the effort and resources needed is an area, where much more research is needed.*** ***This issue has been explored in more detail in the BMJ Open publication but was not tackled in this paper*** ***. ***
3. Section: General Comment: This does not address my previous concerns about recognition of effort of the original researchers or issues about self-identification by patients, but perhaps that is outside of the scope of the paper. It would helpful to remind the reader that these are key, unresolved issues and point to places where they might be considered further.
***These aspects have been discussed in detail in the BMJ open publication and are outside the scope of this paper.  As suggested, readers are reminded that the points raised  by the reviewer are key unsolved issues and initiatives dealing with these issues are referred to.  ***
**Moderate **

1. Section: Table 1 Text ref: "1.2 Clarify own institution’s requirements for data sharing"Comment: This is pretty vague. I don’t know how to use this row.
***This was split into two subprocesses and a comment was added in the table. The order of 1.2 and 1.3 was reversed. ***
2. Section: Table 1 Text ref: "2.1.2 Check funder requirements for data sharing"Comment: Which takes priority and when? 2.1.2 vs 1.2.
***Certainly a reasonable question but so far no priorities have been defined  and the timely order of processes has only be lightly tackled in the figure. The work is part of ongoing research in the CORBEL project.*** ***A comment about "clarification of legal responsibilities" has been added in 2.1.2. ***
3. Section: Table 1 Text ref: "2. Plan for data sharing, in the context of a specific trial"Comment: When should this be developed? 2.2.2 suggests before the protocol is finalised; but I suspect 2.2.2 would generally be done before 2.2.1. What is the ordering of the rows?
***Correct, the order of 2.2.1 and 2.2.2 has been reversed. ***
4. Section: Table 1Text ref: "3.1 Decide upon strategy for data preparation for sharing"Comment: 3.1.1 and 3.1.2 seem to be in the wrong order.
***We have not changed that because from our viewpoint this seems to be the right order. ***
5. Section: Table 1 Text ref: Section 3 or 4Comment: Somewhere, perhaps, one should advertise the timelines for making data available. It’s unlikely to be during the trial; how long after primary analyses? Useful to manage expectations?
***This is an important issue, which has also been discussed in the BMJ Open paper. We have included a reference to timelines in 3.1. ***
6. Section: Table 1 Text ref: "5.1 Maintain highly granular access control to IPD, that can be changed rapidly"Comment: Changed on what basis?
***We removed the reference to "rapid change" as it seems tob e confusing. ***
7. Section: Table 1 Text ref: "5.5 Provide an expert advisory panel"Comment: Is this a Data Access Committee or something different? Is there independent membership? 
***The reference was changed to a Data Access Committee.*** ***We also re-organised the processes in section 5 to make them (I hope) easier to read and understand, though the content is almost exactly the same. 5.3 and 5.4 were split up into sub-processes, 5.5 – 5.7 made subprocesses of a new 5.5, and 5.6 (was 5.8) expanded to include 2 subprocesses of reporting / feedback***
8. Section: Table 1Text ref: "5.7 Provide data use agreement templates"Comment: Possibly wishful thinking. Agreements are never as straightforward as one might hope. Is this a suggestion for global templates, institution templates or trial templates?
***We agree that in practice there will be no agreed templates.*** ***Therefore we added a phrase that the templates may be starting points for negotiated, specific agreements.***
9. Section: Table 1 Text ref: "6.1.2 Assess the reasonableness of the request and the ability of the requesters to draw sensible conclusions"Comment: Where is the independence in this process? Is there a duty from the sponsor and TMG to work fairly? Who judges what is reasonable?
***Yes, a critical issue. This is the reason why we prefer data sharing via trusted repositories with defined and transparent governance. In 6.1 processes are specified for the use case of access via direct contact with the sponsor/PI. Here an independency of processes is usually not given.***
10. Section: Table 1 Text ref :: "6.2.1 Repository makes appropriate request forms available on-line"Comment: Why? This will just encourage false positive submissions. Better for applicants to talk to the trial team before getting a form, so the applicant really understands whether the data set is suitable and timely. (Very often, it really won’t be.)
***We are supporting the view that data sharing and re-use should be possible without the (mandatory) involvement of data generators. False positive submission may be reduced if the data available are fully described***. ***According to the suggestions of another reviewer, a relation between data requester and data generator named « optional collaboration » has been added to the figure. In our consensus exercise (BMJ Open paper) we formulated the following recommendation (no. 33) :  « Collaboration between data providers and secondary data users could be an added value in data sharing.*** ***However, it should not be a pre-requisite for data sharing. ». Therefore we marked the relation with « optional ».***
11. Section Table 1 Text refL "7.2 Agree an ID generation scheme for data objects"Comment: Also, what if the same dataset is given to two separate people: does this get the same ID?
***Yes, the ID is fixed with the clinical trial objects.*** ***7.2. is now split into two related subprocesses, as is 7.3. 7.1. and 7.5 simplified by removal of subprocess.***
12. Section: Table 1 Text ref: "8. Publishing results of re-use"Comment: Who checks that the secondary use of the data is done well?
***Yes, this is a critical issue.*** ***There is no standard procedure foreseen for this. The best strategy is to make the re-analysis fully open and transparent.*** ***(see 8.1.1). In that case the scientific community (including the data generators) can check the validity of the re-analysis.  Nevertheless, monitoring compliance (in general) is an open issue but not impossible. FDAA Trial Tracker is a good example of monitoring compliance to regulation in trial registry and Ben Goldacre’s group is also chasing  and publishing non-compliance. ***
13. Section: Table 1 Text ref: "8. Publishing results of re-use"Comment: What to do if there is discrepancy in findings between original and subsequent findings? Could undermine trust. Probably needs rows about “dispute” resolution.
***Yes, also very important and difficult to solve.*** ***Replication is very important in science (*** ***https://www.nature.com/news/1-500-scientists-lift-the-lid-on-reproducibility-1.19970*** ***) and given that the replication of complex and expensive experiments such as trials is not very much feasible, replication of the analysis is fundamental.*** ***We cannot think of any formal structure, to ‘referee’ disputes, that would be applicable here – any dispute would need to be played out in the literature, and each is likely to have different characteristics. We have restructured section 9 to add a row about the need to monitor disputes, as well as other possible consequences.***
14. Section: Table 2 Text ref: "2. Locator services. Locator service for data sharing resources"Comment: Will this be a familiar term to readers? I’m not sure what it means.
***We have tried to reword section 2 to make the meaning clearer.*** 
**Trivial/Minor **

1. Section: Table 1 Comment: Would be quickly for each actor to find the role if this column was broken into separate columns, one per actor type, with the ticks for whether it is relevant. 
***Table 1 ordered according to actor is an interesting proposal but according to our approach (list all processes/sub-processes following the clinical workflow) it would mean to add another table. We would not prefer to do that to keep the paper as  simple as possible.***
2. Section: Table 1 Text ref: "7.2 Agree an ID generation scheme for data objects"Comment: “Data objects” needs a clear definition before the table. Perhaps a Glossary with the Abbreviations?
***Yes, a glossary with some main terms (defined as used in this paper) was added at the bottom of table 1.***

# Peer review: Referee response for version 1

**DOI:** 10.5256/f1000research.14988.r31016

**Published:** 2018-03-01

**Authors:** Florian Naudet

**Affiliations:** 1 CHU Rennes, Inserm, CIC 1414 (Centre d'Investigation Clinique de Rennes), University of Rennes 1, Rennes, France

The manuscript Classification of processes involved in sharing individual participant data from clinical trials by Ohmann C, Canham S, Banzi R, Kuchinke W and Battaglia S ^1^ is more than useful for all stakeholders interested in data sharing. It must be accepted with, in my opinion, a few (and minor) edits. 

 In my experience as a researcher interested in the impact of data sharing policies ^2^, I have identified that a major practical barrier to implementation of full data sharing of randomised controlled trials was the great heterogeneity across different trial groups: "getting prepared and preplanning for data sharing still seems to be a challenge for many trial groups; data sharing proved to be novel for some authors who were unsure how to proceed". Therefore the description and classification of processes involved in sharing IPD from clinical trials will surely helps all stakeholders to get prepared. It is welcome and this manuscript will be very useful. 

 I have a few suggestions that may help to write it better. Please note that I'm not an expert in qualitative research. Therefore these are only suggestion that I don't want to enforce strongly. 

 First, as it is presented as a research paper and because it is very qualitative by nature, I would suggest to use, or better adapt the reporting guidelines for qualitative research ^3^ to this specific paper as most points won't directly apply since the study presented is not a typical qualitative research. 

 More specifically, I would welcome more details on authors in the main text: 

 - Who are they? Were they from different background (e.g. data managers, statisticians, trialists, patients, etc..., Master degree, MD, PhD, PharmD... etc.). Please clearly state that they were involved in the initial initiative that was used for this paper ^4^. Please also detail how it could have affected their judgement. 

 - What is their background for conducting such a qualitative synthesis?

 - Was there a protocol registered for this analysis? 

 Please specify why the processes were derived from only one initiative ^4^ and not from a systematic assessment of other papers/initiatives. Any limitations of the initial paper should be discussed here.

 The process of analysis should be made as transparent as possible. How the different authors were involved in the process? Were there some leaders during the phone meetings? Were verbatim from written correspondence used? Was there a good agreement between expert (for what parts the agreement was less good ?)? The researchers’ own position should also clearly be stated. A critical examination of their own role, possible bias, and influence on the research would be welcome. 

 I have also identified very practical points that could be addressed in a new version of the manuscript: 

 - In my very practical experience ^2^, figure 1 could be overly simple for being accurate. I think that one important point was missed. Adoption of data sharing in biomedical research not only implies to provide and re-use the data. It implies to adopt a collaborative approach. It means that when one want to re-use the data of another team, one sometimes must directly contact the other team to have information and to have the data in the appropriate format. Sharing data for a re-analysis of safety outcomes involves sharing the cases report forms while re-using data for some IPD meta-analysis may only rely on sharing data at a later analytical stage (e.g. analysable data). This implies that step 3 is very linked with step 6. I think that the figure will be better (if it is not too complex) by adding such kind of relationship. 

 - Table 1, section 1.1.1 / 2.3.1: patients are an important actors/leverages and must be involved in my opinion in these aspects ;

 - Table 1, in general avoid abbreviations such as "SOP" in 1.3 ; 

 - Table 1, section 2 and 3.1: Ethic committees have a strong role to play at all these parts. They have, in my opinion to judge wether the de-identification plan is adapted to the specific study ; 

 - Table 1, section 3.2.1: data manager and statisticians must ensure that the code that will be shared works for the de identified data sets. Practical finding from my experience (in one case, de-identification was made after the analysis and labels were different between the two datasets : therefore the shared code didn't worked).

 - Table 1, section 4.1.1: this should be explored before in my opinion (at step 3), when one decide of the data sharing plan. 

 - Table 2 very interesting, but I would suggest to add an hyperlink to some concrete examples when possible in section 3.

 In general the tables should be checked for majuscule and minuscule: eg. table 2, section 3 "during" must be During. 

 A last suggestion would be to add more practical information for clinicians and to cite the ICJME recommandations. 

 It is again a very great manuscript and I hope that these comment will be able to improve it. 

 I'm not competent to review the English, and please excuse my English.

I have read this submission. I believe that I have an appropriate level of expertise to confirm that it is of an acceptable scientific standard.

## References

[1] OhmannCCanhamSBanziRKuchinkeWBattagliaS: Classification of processes involved in sharing individual participant data from clinical trials. *F1000Research*.2018;7: 10.12688/f1000research.13789.1 10.12688/f1000research.13789.1 PMC586151729623192

[2] NaudetFSakarovitchCJaniaudPCristeaIFanelliDMoherDIoannidisJPA: Data sharing and reanalysis of randomized controlled trials in leading biomedical journals with a full data sharing policy: survey of studies published inThe BMJandPLOS Medicine. *BMJ*.2018;360:k400 2944006610.1136/bmj.k400PMC5809812

[3] TongASainsburyPCraigJ: Consolidated criteria for reporting qualitative research (COREQ): a 32-item checklist for interviews and focus groups. *Int J Qual Health Care*.2007;19(6) : 10.1093/intqhc/mzm042 349-57 10.1093/intqhc/mzm042 17872937

[4] OhmannCBanziRCanhamSBattagliaSMateiMAriyoCBecnelLBiererBBowersSClivioLDiasMDrumlCFaureHFennerMGalvezJGhersiDGluudCGrovesTHoustonPKaramGKalraDKnowlesRLKrleža-JerićKKubiakCKuchinkeWKushRLukkarinenAMarquesPSNewbiggingAO'CallaghanJRavaudPSchlünderIShanahanDSitterHSpaldingDTudur-SmithCvan ReuselPvan VeenEBVisserGRWilsonJDemotes-MainardJ: Sharing and reuse of individual participant data from clinical trials: principles and recommendations. *BMJ Open*.2017;7(12) : 10.1136/bmjopen-2017-018647 e018647 10.1136/bmjopen-2017-018647 29247106PMC5736032

# Response

**Published:** 2018-04-16

**Authors:** Christian Ohmann

**Affiliations:** ECRIN, Germany

***Response to the reviewer in bold and italics***

The manuscript Classification of processes involved in sharing individual participant data from clinical trials by Ohmann C, Canham S, Banzi R, Kuchinke W and Battaglia S ^1^ is more than useful for all stakeholders interested in data sharing. It must be accepted with, in my opinion, a few (and minor) edits. 

In my experience as a researcher interested in the impact of data sharing policies ^2^, I have identified that a major practical barrier to implementation of full data sharing of randomised controlled trials was the great heterogeneity across different trial groups: "getting prepared and preplanning for data sharing still seems to be a challenge for many trial groups; data sharing proved to be novel for some authors who were unsure how to proceed". Therefore the description and classification of processes involved in sharing IPD from clinical trials will surely helps all stakeholders to get prepared. It is welcome and this manuscript will be very useful. 

I have a few suggestions that may help to write it better. Please note that I'm not an expert in qualitative research. Therefore these are only suggestion that I don't want to enforce strongly. 

First, as it is presented as a research paper and because it is very qualitative by nature, I would suggest to use, or better adapt the reporting guidelines for qualitative research ^3^ to this specific paper as most points won't directly apply since the study presented is not a typical qualitative research. 

More specifically, I would welcome more details on authors in the main text: 

- Who are they? Were they from different background (e.g. data managers, statisticians, trialists, patients, etc..., Master degree, MD, PhD, PharmD... etc.). Please clearly state that they were involved in the initial initiative that was used for this paper ^4^. Please also detail how it could have affected their judgement. 

- What is their background for conducting such a qualitative synthesis?

- Was there a protocol registered for this analysis? 

Please specify why the processes were derived from only one initiative ^4^ and not from a systematic assessment of other papers/initiatives. Any limitations of the initial paper should be discussed here.

The process of analysis should be made as transparent as possible. How the different authors were involved in the process? Were there some leaders during the phone meetings? Were verbatim from written correspondence used? Was there a good agreement between expert (for what parts the agreement was less good ?)? The researchers’ own position should also clearly be stated. A critical examination of their own role, possible bias, and influence on the research would be welcome. 

***We agree with the reviewer that this paper can be classified as qualitative research, although we applied a semi-formal collaborative small group decision-making approach and not a formal methodology such as interviews or focus groups.*** ***We revised the manuscript and adapted it as much as possible to the COREQ guidelines.. However, as expected, many COREQ items are clearly not applicable. We hope this revision had improved the paper reporting.***

I have also identified very practical points that could be addressed in a new version of the manuscript: 

- In my very practical experience ^2^, figure 1 could be overly simple for being accurate. I think that one important point was missed. Adoption of data sharing in biomedical research not only implies to provide and re-use the data. It implies to adopt a collaborative approach. It means that when one want to re-use the data of another team, one sometimes must directly contact the other team to have information and to have the data in the appropriate format. Sharing data for a re-analysis of safety outcomes involves sharing the cases report forms while re-using data for some IPD meta-analysis may only rely on sharing data at a later analytical stage (e.g. analysable data). This implies that step 3 is very linked with step 6. I think that the figure will be better (if it is not too complex) by adding such kind of relationship. 

***According to the suggestions of the reviewer, a relation between data requester and data generator named « optional collaboration » has been added to the figure. In our consensus exercise (BMJ Open paper)  we formulated the following recommendation (no. 33) :  « Collaboration between data providers and secondary data users could be an added value in data sharing. However, it should not be a pre-requisite for data sharing. ». Therefore we marked the relation with « optional ». *** 

- Table 1, section 1.1.1 / 2.3.1: patients are an important actors/leverages and must be involved in my opinion in these aspects ;

***Added patient groups to list of actors for 1.1.1, 2.3.1 and 2.3.2***

- Table 1, in general avoid abbreviations such as "SOP" in 1.3 ; 

***A brief definition has been added to the glossary of at the bottom of table 1..***

- Table 1, section 2 and 3.1: Ethic committees have a strong role to play at all these parts. They have, in my opinion to judge wether the de-identification plan is adapted to the specific study ; 

***We are not sure if the exact role of ethics committees in data sharing has been clarified, though if the proposals are in the protocol and the participant information sheet (etc.) they would be scrutinised by an ethics committee.  Not sure if this needs to be added explicitly as part of the workflow unless ECs are given a formal role.***

- Table 1, section 3.2.1: data manager and statisticians must ensure that the code that will be shared works for the de identified data sets. Practical finding from my experience (in one case, de-identification was made after the analysis and labels were different between the two datasets : therefore the shared code didn't worked).

***An extra subprocess has been added as 3.2.2.***

- Table 1, section 4.1.1: this should be explored before in my opinion (at step 3), when one decide of the data sharing plan. 

***We are not so sure. This will never be a simple linear process, so the order in the table does not imply a similar ordering of workflow. We have changed 4.1. so that it is either a selection or a confirmation of an earlier repository selection.***

- Table 2 very interesting, but I would suggest to add an hyperlink to some concrete examples when possible in section 3.

***Table 1 and 2 were improved, taken the comments from the reviewer into consideration.***

In general the tables should be checked for majuscule and minuscule: eg. table 2, section 3 "during" must be During.

***Checked.***

A last suggestion would be to add more practical information for clinicians and to cite the ICJME recommandations. 

***The activity  of ICMJE was cited.***

It is again a very great manuscript and I hope that these comment will be able to improve it. 

I'm not competent to review the English, and please excuse my English.
